# Deletion of platelet-derived growth factor receptor β suppresses tumorigenesis in metabolic dysfunction-associated steatohepatitis (MASH) mice with diabetes

**DOI:** 10.1038/s41598-024-75713-6

**Published:** 2024-10-11

**Authors:** Tsutomu Wada, Yuki Takeda, Akira Okekawa, Go Komatsu, Yuichi Iwasa, Yasuhiro Onogi, Ichiro Takasaki, Takeru Hamashima, Masakiyo Sasahara, Hiroshi Tsuneki, Toshiyasu Sasaoka

**Affiliations:** 1 Department of Clinical Pharmacology, University of Toyama, 2630 Sugitani, Toyama, 930-0194 Japan; 2 Research Center for Pre-Disease Science, University of Toyama, 2630 Sugitani, Toyama, Japan; 3 Department of Pharmacology, Graduate School of Science and Engineering, University of Toyama, 3190 Gofuku, Toyama, Japan; 4 Department of Pathology, University of Toyama, 2630 Sugitani, Toyama, Japan; 5 Department of Integrative Pharmacology, University of Toyama, 2630 Sugitani, Toyama, Japan

**Keywords:** Hepatic stellate cells (HSC), MicroRNA, Metabolic dysfunction-associated steatohepatitis (MASH), Platelet-derived growth factor (PDGF), Diseases, Endocrinology

## Abstract

The platelet-derived growth factor (PDGF) family contributes to the progression of steatohepatitis; however, changes in and the characteristics of isoform-specific expression remain unclear. Since diabetes is a major driver of metabolic dysfunction-associated steatohepatitis (MASH), we characterized the mouse model of diabetic MASH (dMASH) by focusing on PDGF signaling. *Pdgfa-d* expression was markedly higher in hepatic stellate cells among flow-sorted cells in control mice and also increased in dMASH. In contrast, a reanalysis of human single-cell RNA-Seq data showed the distinct distribution of each PDGF isoform with disease progression. Furthermore, inflammation and fibrosis in the liver were less severe in diabetic MASH using tamoxifen-induced PDGF receptor β (PDGFRβ)-deficient mice (KO) than in control dMASH using floxed mice (FL) at 12 weeks old. Despite the absence of tumors, the expression of tumor-related genes was lower in KO than in FL. Tumorigenesis was significantly lower in 20-week-old KO. An Ingenuity Pathway Analysis of differentially expressed miRNA between FL and KO identified functional networks associated with hepatotoxicity and cancer. Therefore, PDGFRβ signals play important roles in the progression of steatohepatitis and tumorigenesis in MASH, with the modulation of miRNA expression posited as a potential underlying mechanism.

**Supplementary Information:**

The online version contains supplementary material available at 10.1038/s41598-024-75713-6.

## Introduction

Liver cancers, with hepatocellular carcinoma (HCC) accounting for most cases, are the fourth most common cause of cancer-related death. The prevalence of metabolic dysfunction-associated steatohepatitis (MASH), formerly known as nonalcoholic steatohepatitis (NASH), is increasing. MASH has emerged as a leading cause of HCC with the widespread availability of effective antiviral drugs against hepatitis B and C viruses in recent years^1^. Diabetes increases the risk of MASH and HCC^1–3^. Although several MASH-driven HCC mouse models have been established, the development of HCC typically takes a considerable amount of time, approximately one year for most mouse models^2,4^. An excellent model mouse of diabetic steatohepatitis has recently been proposed. Mice are prepared by the administration of streptozotocin (STZ) on the second day after birth and fed a high-fat diet (HFD) from 4 weeks old. Mice exhibit the histological features of MASH in a time-dependent manner. The liver of this model shows hepatic steatosis at 6 weeks old, fibrosis at 8 weeks old, and progresses to HCC at 20 weeks old in most mice^5^. Since this model is relatively easy to generate and develops into HCC in a short period of time, it has been used to analyze steatohepatitis and HCC under diabetic conditions^4^.

Platelet-derived growth factor (PDGF) is a family of growth factors mainly derived from mesenchymal cells and comprises isoforms A-D. Each isoform exhibits unique biological functions by binding to their respective receptors through specific binding properties; PDGF-A and C bind to PDGF receptor (PDGFR)α, PDGF-B binds to PDGFRα and β, and PDGF-D binds to PDGFRβ^6^. They play a wide range of roles in various physiological and pathological aspects, such as vascular development and angiogenesis, cancer proliferation, metastasis, invasion, wound healing, and the promotion of fibrosis^6–9^. Hepatic stellate cells (HSCs) are the principal profibrotic cells in the liver, and PDGFRβ signaling is one of the most potent mediators promoting hepatic fibrosis^10,11^. In contrast, PDGF-A/PDGFRα signaling is considered to play important roles in the progression of HCC^8,12^. Recent findings from single-cell RNA-Seq analyses revealed the pathological significance of cell-cell interactions between HSCs, endothelial cells (ECs), Kupffer cells (KCs), macrophages (MFs), and damaged hepatocytes in driving the progression of hepatic fibrosis and carcinogenesis in MASH^13–17^; however, the expression profiles of PDGFs and their receptors in individual cells remain unclear, particularly in the development of MASH with diabetes. Furthermore, although miRNAs appear to be closely associated with the progression of MASH^18–20^, the effects of PDGF signaling on miRNA expression profiles in diabetic MASH models remain unclear.

Therefore, the present study investigated the pathological role of PDGFRβ signaling in the development of hepatic fibrosis and tumor formation in a diabetic MASH model. We isolated HSCs, ECs, KCs, and MFs from the liver at the fibrosis stage in a diabetic MASH model using C57BL/6J mice. We then characterized the expression profiles of the *Pdgf* family in these cells. We also examined the impact of the deletion of PDGFRβ on hepatic fibrosis and tumor formation in the current diabetic MASH model. Furthermore, we investigated the expression profiles of miRNAs potentially involved in the progression of MASH in non-tumor areas of the livers of PDGFRβ knockout mice.

## Results

### Characterization of diabetic MASH mice on the C57BL/6J background

**Fig. 1:**
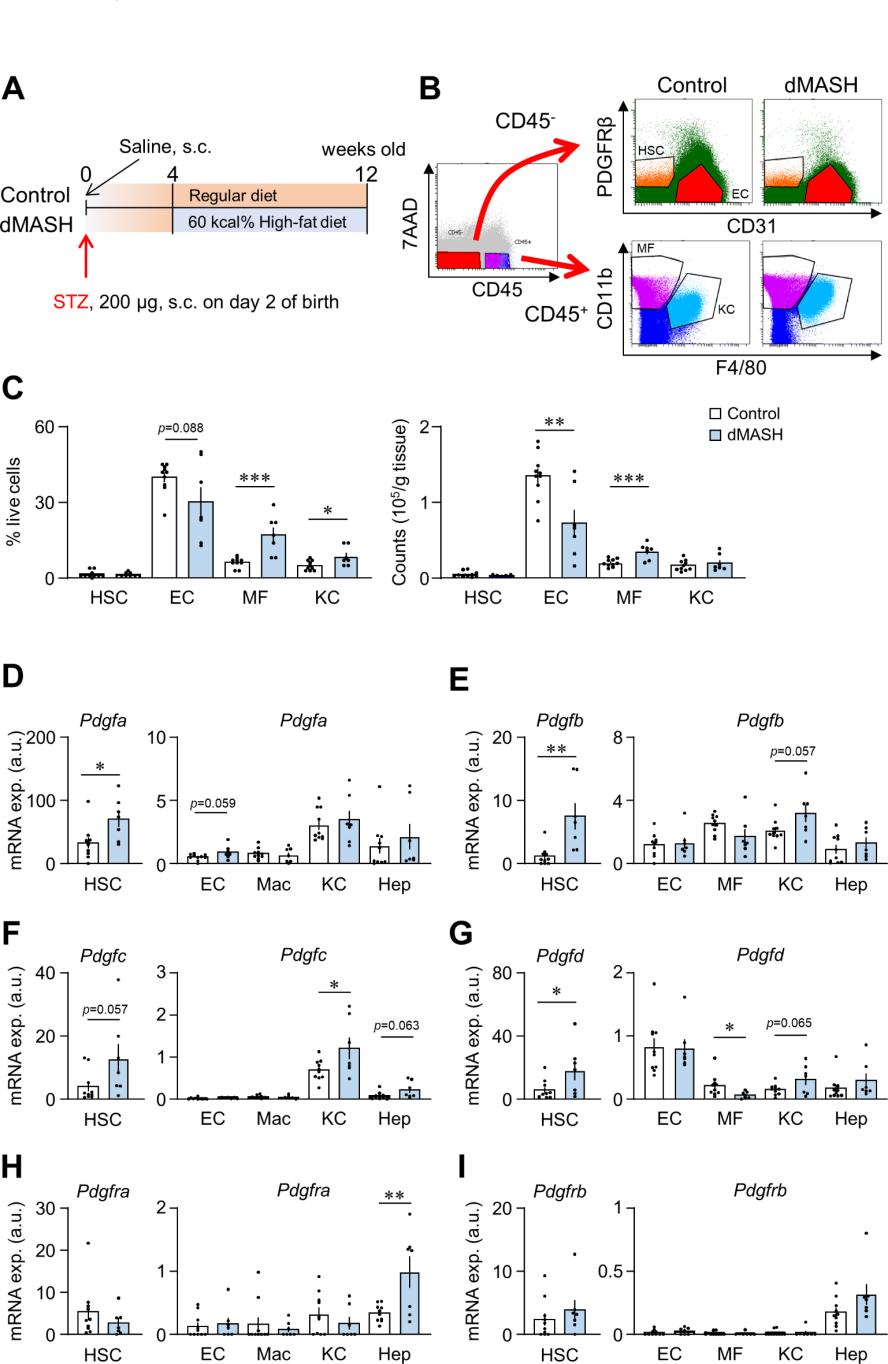
Changes in cell populations and expression profiles of thePdgf family in diabetic MASH mice on the C57BL/6J background. (**A**) Schema of the protocol for generating diabetic MASH (dMASH) in C57BL/6j mice. (**B**) Gating of liver non-parenchymal cells. CD45^−^PDGFRβ^+^ cells, CD45^−^CD31^+^ cells, CD45^+^F4/80^lo^CD11^hi^ cells, and CD45^+^F4/80^hi^CD11b^lo^ cells in liver non-parenchymal cells were isolated as hepatic stellate cells (HSC), endothelial cells (EC), Kupffer cells (KC), and infiltrated macrophages (MC), respectively. (**C**) The ratios and absolute cell numbers of each cell in control and dMASH livers. (**D-I**) mRNA levels of *Pdgf* and the *Pdgfr* family in each cell. Data are presented as the mean ± SEM (Control, *n* = 10; dMASH, *n* = 7). *p < 0.05 and **p < 0.01.

We examined the characteristics of diabetic MASH model mice generated using C57BL/6J mice (dMASH), as shown in Fig. 1A, which exhibit steatohepatitis at 12 weeks old (Supplementary Fig. S1)^5^. Blood glucose levels were significantly higher in dMASH than in control C57BL/6J mice. Body weights were lower and liver weights were higher in dMASH. The appearance of the livers of dMASH was swollen and whitish in color, and the typical histological changes of MASH, such as lipid accumulation in hepatocytes and the infiltration of immune cells, were observed in hematoxylin and eosin (H&E) staining. The NAFLD activity score (NAS), a histological scoring system for evaluating steatohepatitis^21^, was close to zero in control livers, whereas it was significantly higher in dMASH livers. In addition, Sirius-Red staining revealed hepatic fibrosis in dMASH livers. Consistent with histological findings, the expression of the proinflammatory genes *Emr1*, encoding F4/80, and *Tnfa* as well as fibrosis-related genes was significantly higher in dMASH livers than in control livers. Moreover, the expression of *Pdgfa*,* b*,* c*, as well as *Pdgfra* and *b* was higher in dMASH livers (Supplementary Fig. S1).

We characterized the expression profiles of *Pdgf* isoforms in the non-parenchymal cells (NPCs) of dMASH livers. We isolated hepatocytes with Percoll, and liver NPCs were flow-sorted into HSCs, ECs, MFs, and KCs. The gating strategy for NPCs is shown in Fig. 1B. The percentages of MFs among NPCs were significantly higher in dMASH livers than in control livers. The proportion of KCs, which is known to decrease in MASH and MASH animal models^22^, unexpectedly increased. In a comparison of actual cell numbers, a significantly lower number of ECs and higher number of MFs were observed in dMASH livers than in control livers (Fig. 1C). In these cells, the expression of *Pdgf* isoforms was markedly higher in HSCs than in other cell types, with *Pdgfa*, *b*, *d* significantly increasing and *Pdgfc* slightly increasing in the HSCs of dMASH livers. In cells other than HSCs, *Pdgfc* significantly and slightly increased in KCs and hepatocytes, respectively. In contrast, *Pdgfd* decreased in MFs and slightly increased in KCs (Fig. 1D-G). Interestingly, the expression of *Pdgfra* was increased in damaged hepatocytes in dNASH, whereas no significant changes in receptor expression were observed in other cells (Fig. 1H and I, Fig. S2B).

**Fig. 2:**
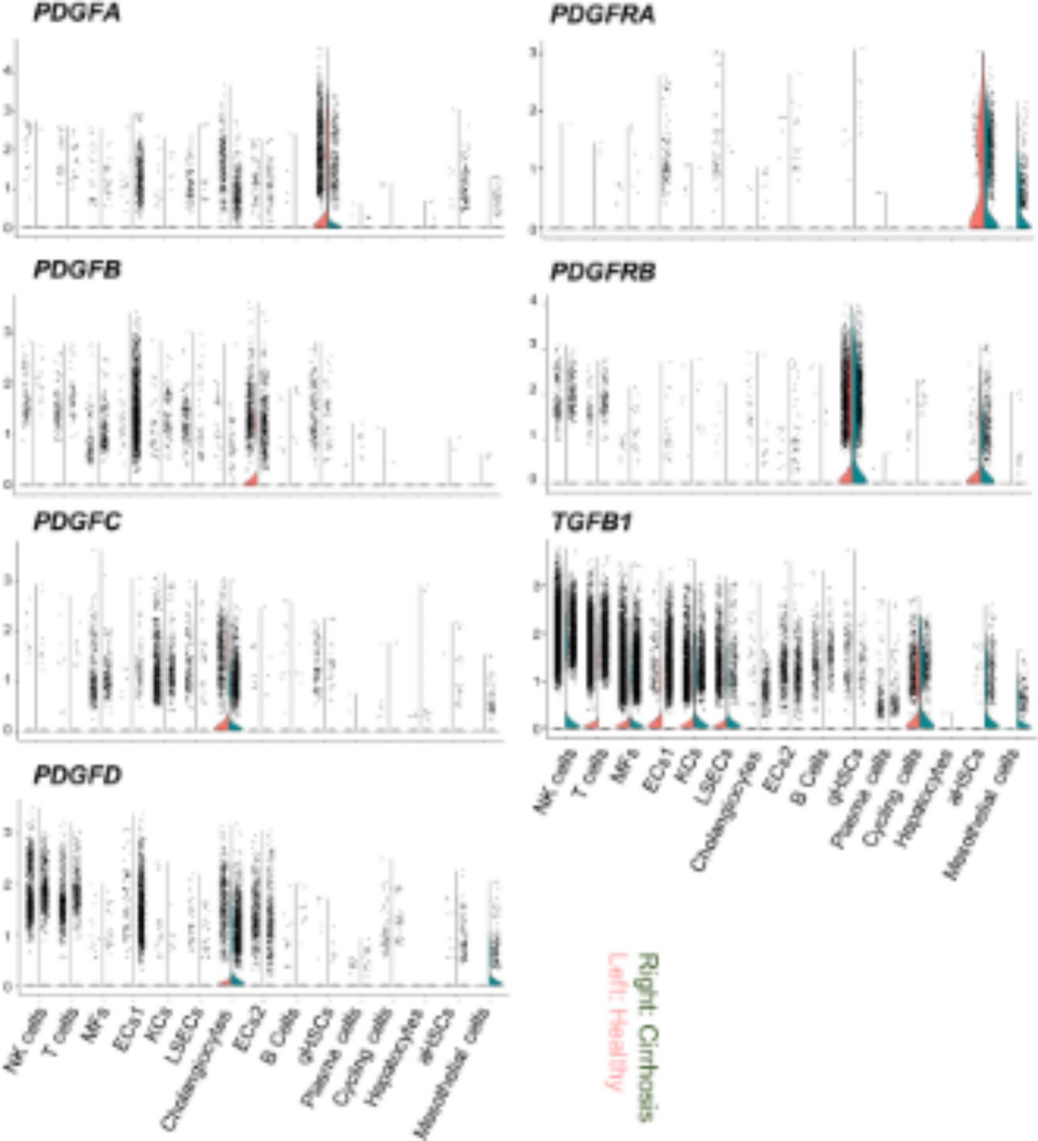
Expression profiles of the PDGF family in healthy subjects and cirrhosis patients. Violin plot of PDGF-related gene expression by cell type reanalyzed using public scRNA-Seq data. The left and right columns show the results for healthy subjects and cirrhosis patients, respectively.

We then investigated the expression profiles of PDGF isoforms in the liver NPCs of healthy and cirrhosis subjects using publicly available single-cell RNA-seq (scRNA-Seq) data^13,16^. We analyzed the expression of PDGF isoforms and their receptors, as the profibrotic factor TGFB1, in 15 annotated cell populations, including NK cells, T cells, B cells, Plasma cells, MFs, 2 subclusters of ECs, liver sinusoidal ECs (LSECs), cholangiocytes, quiescent and active HSCs, cycling cells, hepatocytes, and mesothelial cells (Fig. 2, Supplementary Fig. S2). *PDGFA* was predominantly expressed in quiescent HSCs (qHSCs) but is also expressed in ECs subpopulation 1 (ECs1) and activated HSCs (aHSCs), and slightly expressed in mesothelial cells in cirrhotic livers. In contrast, *PDGFB* was mainly expressed in ECs, and increased and slightly increased in ECs1 and KCs, respectively, in cirrhosis livers. On the other hand, *PDGFC* and *PDGFD* were predominantly expressed in cholangiocytes, ECs, and some immune cells, and a slight increase in *PDGFD* was observed in aHSCs and mesothelial cells in cirrhotic livers. *TGFB1*, a fibrosis-promoting gene, was expressed in many types of cells, and mainly increased in ECs1, aHSCs, and mesothelial cells in cirrhotic livers (Supplementary Fig. S2).

### Impact of the deletion of PDGFRβ on hepatic fibrosis and steatohepatitis in dMASH

**Fig. 3:**
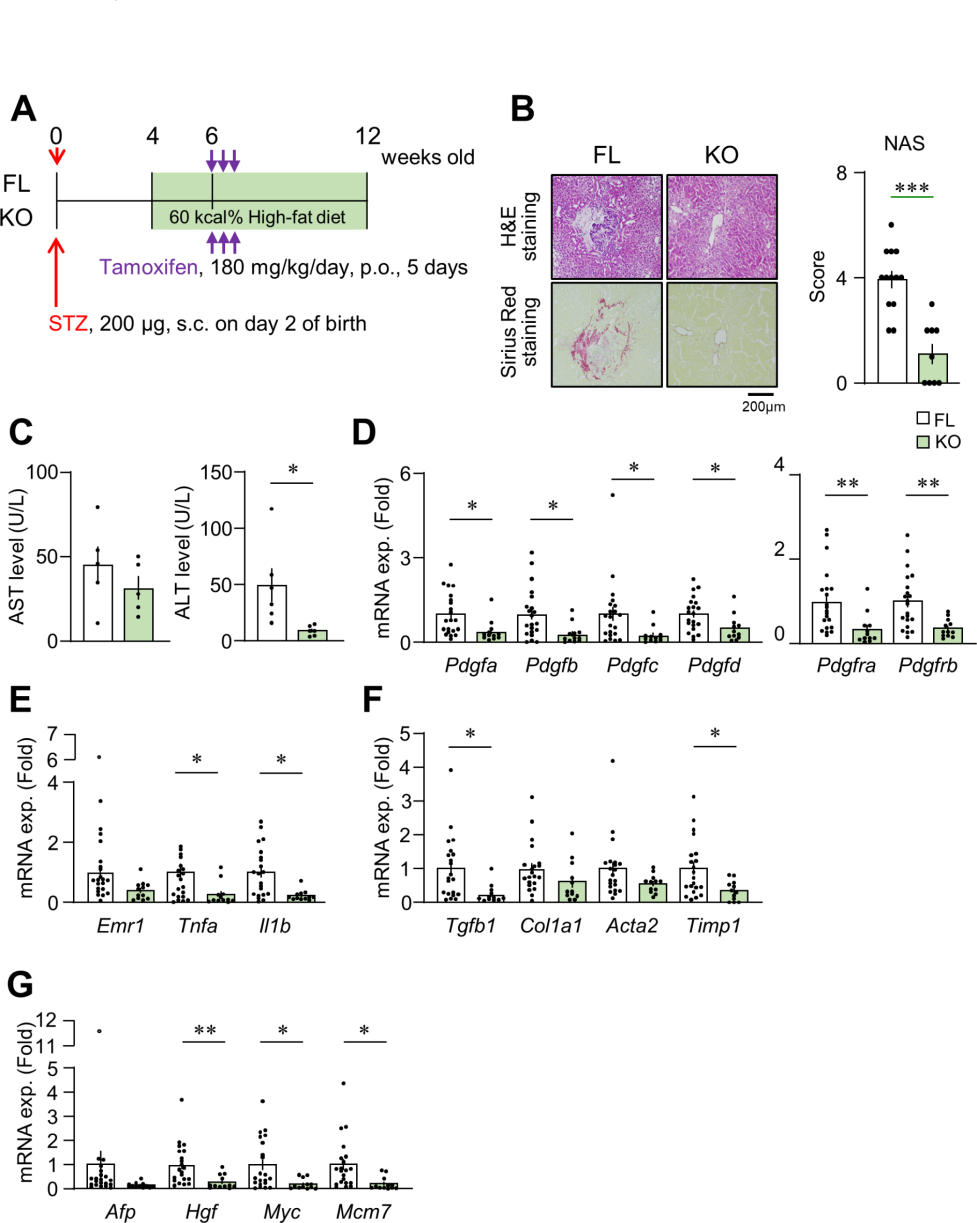
Impact of the deletion of PDGFRβ on steatohepatitis in dMASH at 12 weeks old. (**A**) Schema of the protocol for producing PDGFRβ-deficient dMASH. KO and FL mice were orally administered tamoxifen for 5 consecutive days at 6 weeks old to achieve the knockout of *pdgfrb*. Mice were analyzed at 12 weeks old. (**B**) Representative photomicrographs of the liver in H&E and Sirius-Red staining, and a histological evaluation by NAS (FL, *n* = 13; KO, *n* = 9). Scale bar = 200 μm. (**C**) Serum AST and ALT levels (*n* = 5–6). (**D-G**) mRNA levels of *Pdgf* isoform-, inflammation-, fibrosis-, and tumorigenesis-related genes. Data are presented as the mean ± SEM (FL, *n* = 21; KO, *n* = 12). *p < 0.05, **p < 0.01, and ***p < 0.001.

The impact of the deletion of *Pdgfrb* on steatohepatitis was examined in 12-week-old diabetic MASH mice with systemic *Pdgfrb* knockout (KO) and control (FL) backgrounds (Fig. 3A). KO and FL showed similar elevations in blood glucose levels and changes in body weights, but no significant difference in liver weights at the time of dissection (Supplementary Fig. S3). Similar to dMASH in C57BL/6J mice (Supplementary Fig. S1), FL livers exhibited the histological features of MASH, including inflammation and fibrosis, whereas inflammation was ameliorated and fibrosis was mostly absent in KO livers. NAS was lower in KO than in FL (Fig. 3B, Supplementary Fig. 3D). Serum AST levels remained unchanged, whereas serum ALT levels were significantly lower in KO than in FL (Fig. 3C). The expression of each *Pdgf* isoform, as well as *Pdgfra* and *Pdgfrb*, was significantly lower in KO livers than in FL livers (Fig. 3D). In addition, the expression of the proinflammatory genes *Tnfa* and *Il1b* was significantly lower, while that of *Emr1* was slightly lower in KO livers (Fig. 3E). Regarding fibrosis-related genes, the expression of *Tgfb1* and *Timp1* was lower in KO, while that of *Col1a1* and *Acta2* was similar between FL and KO (Fig. 3F). The expression of the carcinoma-related genes *Hgf*, *Myc*, and *Mcm7* was lower in KO livers than in FL livers, despite the absence of detectable tumor lesions in the liver upon histological examination at 12 weeks old (Fig. 3G).

### Impact of the deletion of PDGFRβ on tumor progression in dMASH livers

**Fig. 4:**
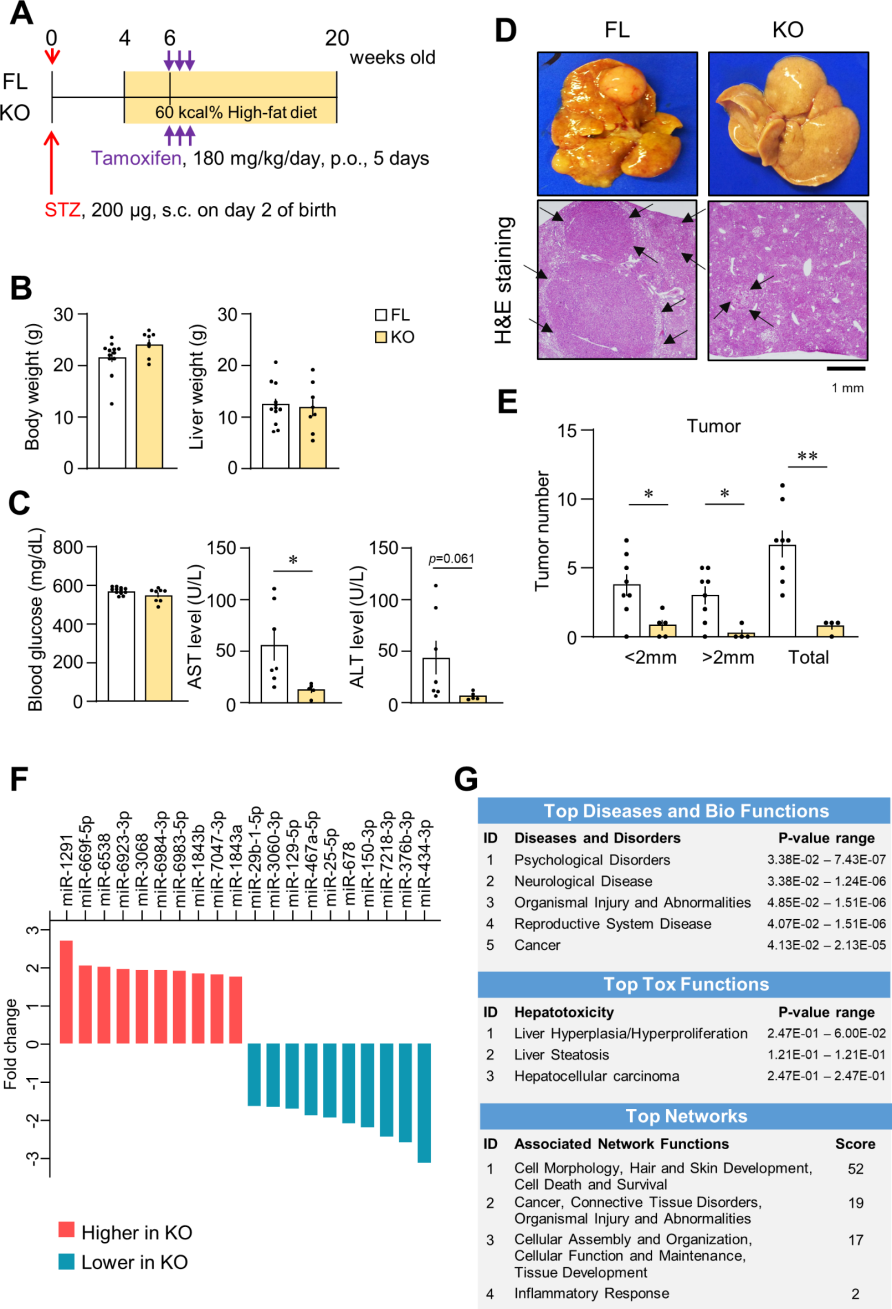
Impact of the deletion of PDGFRβ on tumorigenesis and progression in dMASH at 20 weeks old. (A) Schema of the protocol for producing PDGFRβ-deficient dMASH. Mice were analyzed at 20 weeks old. (B) Body and liver weights of mice (FL, *n* = 12; KO, *n* = 8). (C) Serum glucose (FL, *n* = 12; KO, *n* = 8) and AST and ALT levels (FL, *n* = 7; KO, *n* = 5). (D) Macroscopic appearance of the whole liver and a representative photomicrograph of a liver section in H&E staining. Arrows indicate the edges of tumors. Scale bar = 1 mm. (E) Total numbers of tumors and numbers of tumors by size in the entire left lateral, left median, and caudate lobes of FL and KO livers (FL, *n* = 8; KO, *n* = 4). Data are presented as the mean ± SEM. *p < 0.05 and **p < 0.01. (F) The top 10 up- and down-regulated miRNAs in the non-tumor regions of the livers of 20-week-old KO relative to FL (FL, *n* = 5; KO = 5). (G) Ingenuity Pathway Analysis (IPA) of miRNAs showing expression changes in KO livers. Significant functions identified in KO are listed according to their *p*-value.

We investigated the impact of the PDGFRβ deletion on tumor progression in dMASH by analyzing 20-week-old FL and KO mice (Fig. 4A). Body and liver weights and blood glucose levels were similar between FL and KO. However, serum ALT levels were slightly lower and serum AST levels were significantly lower in KO than in FL (Fig. 4B and C). Macroscopic and microscopic observations revealed many tumors in FL livers and significantly fewer tumors in KO livers (Fig. 4D). In an analysis of 2-mm-thick sections across the left lateral, left median, and caudate lobes of the liver, the total tumor number and the numbers of tumors ≥ 2 mm and < 2 mm were significantly lower in KO than in FL (Fig. 4E). Despite the difference in tumorigenesis, the mRNA expression of *pdgf* isoforms was similar in tumor and non-tumor areas in both FL and KO livers at 20 weeks old. Although PDGFRβ expression in KO mice was significantly decreased at 12 weeks old (Fig. 3D), the difference disappeared in both tumor and non-tumor areas at 20 weeks of age (Fig. S4). This may be due to the emergence of newly induced PDGFRβ and PDGFRβ-expressing cells as MASH progresses following tamoxifen administration. The expression of *Afp*, a typical tumor marker for HCC, was higher in tumor lesions than in non-tumor areas in FL. However, other tumorigenesis-related genes that showed higher expression in FL than in KO at 12 weeks old did not significantly differ between tumor and non-tumor areas in both FL and KO livers (Supplementary. Fig. S4). As more detailed analyses, we performed immunohistochemical staining for AFP, c-Myc, and MCM7 (Supplementary. Fig. S5). In the livers of certain FL mice, staining for AFP in the cytoplasm and c-Myc in the nucleus was observed only in limited areas, primarily within tumor lesions, whereas the majority of nodules were negative for the staining in both FL and KO mice. Notably, the cells stained for AFP and c-Myc did not always overlap and were distinct from each other. A similar staining pattern was observed in the livers of some KO mice, although the stained regions were minimal due to the smaller number and size of tumors. These histological distributions may explain the reason that the real-time PCR analysis performed on homogenized tissue did not detect significant differences in *Afp* and *Myc* expression between the two groups (Supplementary Fig. S4). Conversely, MCM7 was robustly stained in the nuclei of numerous hepatocytes within the tumors of both FL and KO mice. Interestingly, while many MCM7-positive cells were also present outside the nodules in the FL liver, the frequency of such cells was considerably lower in the KO liver.

### Impact of the deletion of PDGFRβ on miRNA expression in dMASH livers

Changes in the expression of miRNAs associated with liver injury contribute to the progression of the MASH pathology^18–20^. Therefore, we compared miRNA expression profiles in non-tumor areas in FL and KO livers at 20 weeks old. miRNA expression significantly differed between FL and KO, as shown in volcano plot (Supplementary Fig. S6A). A list of miRNAs showing marked changes in expression and the results of an Ingenuity Pathway Analysis (IPA) of differentially expressed miRNAs are shown (Fig. 4F and G). The expression of miRNA-1291 showed the greatest increase, whereas that of miRNA-434-3p the greatest decrease in KO. Furthermore, IPA identified relationships with “organismal injury and abnormalities” and “cancer” concerning disease and disorders, whereas “liver hyperplasia/hyperproliferation”, “liver steatosis”, and “HCC” were linked to toxic functions. Moreover, four networks were identified in the network analysis of differentially expressed miRNAs (Fig. 4G). Network 1 predominantly participated in processes related to cell morphology and cell death/survival, while network 2 was primarily associated with cancer and organism injury and abnormalities (Fig. 4G, Supplementary Fig. S6B). These results suggest the involvement of changes in the miRNA profiles of KO livers in the attenuation of MASH progression and tumor formation through these networks.

## Discussion

The present study investigated the role of PDGF signaling in the pathology of steatohepatitis and tumorigenesis in MASH associated with diabetes. We examined disease-associated changes in cell populations in the dMASH mouse model using C57BL6/J mice. The number of ECs was lower while that of MFs was higher in dMASH livers than in control livers. Each PDGF isoform was predominantly expressed in HSCs, and their expression significantly increased with the progression of the dMASH pathology. Although they occurred on a small scale, isoform-specific increases or slight increases in the PDGF family were observed in other cell types in the liver, suggesting the contribution of each isoform to the progression of the present diabetic MASH model. The suppression of fibrosis and chronic inflammation was observed in PDGFRβ-deficient dMASH at 12 weeks old. MASH-associated tumorigenesis was significantly attenuated in KO livers at 20 weeks old, and was accompanied by changes in the expression of miRNAs associated with liver steatosis and HCC. Therefore, PDGFRβ signaling and associated changes in miRNA expression may be involved in the development of chronic inflammation and fibrosis as well as subsequent tumorigenesis during the development of diabetic MASH.

Insulin resistance and associated lipotoxicity are the pathophysiological mechanisms responsible for the development and progression of MASH^3^. Glycemic control, rather than weight reduction, is more closely associated with the suppression of liver fibrosis in MASH^23^. Moreover, glycolytic intracellular metabolism associated with hyperglycemia may trigger transcriptional activation, leading to inflammatory gene expression, along with the up-regulated expression of PDGF isoforms^24–26^. Conversely, our previous findings indicated that neither a PDGF-B stimulation nor the deletion of PDGFRβ affected the expression of lipopolysaccharide-induced inflammatory cytokines in bone marrow-derived MFs^7,26^. The progression of the MASH pathology is promoted by diverse cell-cell interactions, including chronic inflammation, sustained tissue damage, and PDGF signaling^13–15^. Consequently, the attenuation of chronic inflammation and fibrosis observed in the livers of PDGFRβ-deficient diabetic MASH mice (Fig. 3) was attributed to a decline in these interactions in HSCs and activated mesenchymal cells rather than the execution of a direct anti-inflammatory effect in KCs and MFs. A reanalysis of human single cells showed that *PDGFRB* expression was highly expressed in qHSCs, while it was slightly expressed in aHSCs of healthy livers. However, the expression remained high in aHSCs of the liver of cirrhosis (Fig. 2, Fig. S2). Therefore, we speculate that the suppression of steatohepatitis in KO mice is mainly due to the reduction of PDGFRβ signaling in qHSCs, which attenuates the transition of qHSCs to aHSCs. In advanced MASH, the reduction of PDGFRβ signaling in aHSCs, may prevent further pathological progression to tumor formation in KO mice.

A single-cell analysis of the livers of diabetic MASH model mice using carbon tetrachloride was recently performed^27^. In the model, diabetic conditions led to an increase in neutrophils and Ly6C^hi^ MFs and a decrease in LSECs and KCs. The present results are consistent with these findings (Fig. 1), and the utilization of scRNA-Seq enables an analysis of composition ratios and transcriptomes across a broader spectrum of cell types. A reduction in LSECs, along with the transcriptomic transition of LSECs in zone 3 near the central vein to capillary ECs, has been identified as a mechanism contributing to the progression of liver cirrhosis^28^. A reanalysis of publicly available human data revealed differences in the expression profiles of PDGF isoforms between humans and mice. Specifically, cholangiocytes emerged as the likely source of PDGF-C and -D production in human MASH (Fig. 2). Since the significance of cholangiocytes in MASH development has been reported in mice^8^, further investigations, possibly using a scRNA-Seq analysis, are needed to facilitate our understanding of the mechanisms by which the deletion of PDGFRβ suppresses the progression of MASH with diabetes.

The PDGF-B and -D/PDGFRβ axis plays crucial roles in promoting hepatic fibrosis^10,13,14^. Recent studies demonstrated that mice deficient in PDGFRα specifically within HSCs or hepatocytes exhibited diminished fibrosis in model mice with drug-induced liver injury^29,30^, suggesting the involvement of PDGFRα signaling in hepatic fibrosis. PDGFRα signaling is also known to participate in liver tumorigenesis^6,12,31^. The overexpression of PDGF-C, which binds to PDGFRα, but not PDGFRβ, induced HCC in mice^32^. In contrast, expression of not only PDGFRα but also PDGFRβ is known to be a poor prognostic factor in human HCC^8^. However, PDGFRβ in HSCs did not affect carcinogenesis induced by a combined treatment with diethylnitrosamine and carbon tetrachloride^11^. It remained unclear whether PDGFRβ signaling is involved in MASH-associated tumorigenesis. To address this issue, we analyzed the dMASH model using KO mice. In the present study, we demonstrated that the number and size of hepatic tumors decreased in 20-week-old KO (Fig. 4). Therefore, our results indicate that PDGFRβ signaling also participates in liver tumorigenesis during MASH development. In addition, in our dMASH model, an increase in PDGFRα was observed in hepatocytes (Fig. 1H). Thus, the decreased expression of PDGFRα in KO (Fig. 3D) may contribute to the suppression of tumor formation and proliferation in KO. Alternatively, the attenuated progression of steatohepatitis in KO may affect the subsequent reduction in tumorigenesis. This notion is supported by the lower expression levels of *Hgf*, *Myc*, and *Mcm*7 in KO than in FL at 12 weeks old. HGF plays a role in the progression of HCC by promoting the proliferation of tumor-associated fibroblasts (CAFs) in diabetic MASH^33^. In addition, the increase observed in *MCM7* is associated with tumorigenesis and proliferation in human HCC associated with MASH^34,35^. Moreover, PDGF-B has been reported to regulate the transformation of fibroblasts into CAFs and the expression of myc^36,37^. Therefore, the inhibition of PDGFRβ from an early stage of MASH may effectively suppress tumor development by alleviating these pathologies.

MiRNAs have been implicated in the progression of various liver diseases, including MASH^18–20^, and their circulating levels are proposed as potential biomarkers^38^. Since the progression of MASH was suppressed in KO, we conducted a comparative analysis of miRNA expression in non-tumor areas of the liver between 20-week-old FL and KO. KO livers showed changes in the expression profiles of miRNAs functionally associated with steatosis and HCC as identified by IPA (Fig. 4). Among individual miRNAs, miRNA-150, -29, and -129-5p, which decreased in KO, increased in fatty liver disease and type 2 diabetes mellitus (T2DM). They were also involved in fibrosis, insulin resistance, tumorigenesis, and HCC proliferation^18,20,39^. In addition, miR-1291, the expression of which showed the greatest increase in KO, was reported to suppress tumor growth and tumorigenesis in MASH^40^. Furthermore, Gpr55 was identified as one of the targets of miRNA-1291 through a network analysis, as shown in network 2 (Supplementary Fig. S6). Gpr55, a putative cannabinoid receptor, has been associated with the development of MASH via lipogenesis and the activation of HSCs in human subjects^41^. In the present study, we validated that miRNA1291 expression was higher in KO than in FL at both 12 and 20 weeks old, and that *Gpr55* expression was lower in KO than in FL at 12 weeks old in real-time PCR analysis (Supplementary Fig. S6C, D). We did not directly prove the importance of the miRNA1291-Gpr55 pathway for the development of MASH. Nevertheless, we assume that the attenuated progression of steatohepatitis in KO may be associated with the miRNA1291-mediated suppression of Gpr55 expression. In addition to Gpr55, several mRNAs implicated in the development of MASH and tumorigenesis exhibited significant changes between FL and KO mice at 12 weeks old; however, their expression became similar by 20 weeks old (Fig. 3, supplementary Fig. S4). These results suggest that pathological events during the steatohepatitis stage affect subsequent liver tumorigenesis during MASH development. Since MASH progresses with the direct involvement of PDGFRβ-signaling in its pathology^6,8,10,11,13,14,36^, changes in miRNA expression in KO mice may be induced either directly by the loss of PDGFRβ signaling or indirectly by the amelioration of steatohepatitis. The underlying molecular mechanisms by which these changes in miRNA expression in KO mice suppress the MASH pathology need to be elucidated in future studies.

In conclusion, we demonstrated the significance of PDGFRβ signaling across various aspects of MASH progression, including inflammation, steatosis, and liver fibrosis to tumor formation and proliferation. Changes in the expression of miRNAs associated with cancer and hepatotoxicity were observed in dMASH of PDGFRβ KO mice. The present results further reinforce the utility of dNASH model mice in combination with genetically manipulated mice because these mice develop MASH and form tumors within a short period of time. Since the number of MASH patients with diabetes is expected to increase in the future, the present results will contribute to a more detailed understanding of the pathophysiology of and development of novel therapeutics for MASH.

## Methods

### Animals and experimental design

Tamoxifen-induced systemic *Pdgfrb*-KO mice were obtained by breeding *Pdgfrb*^flox/flox^ mice^9^ on the C57BL/6J background with Cre-estrogen receptor transgenic (Cre^ERTM^) mice (Jackson Laboratories, JAX stock #004682), as previously^7,42^. dMASH was generated as reported in a previous study^5^, with minor modifications. In brief, 200 µg of Streptozotocin (STZ) was subcutaneously injected into male C57BL/6J (Japan SLC; Shizuoka, Japan) or genotype-untyped *Pdgfrb*^*f*lox/flox^ and Cre^ERTM^/*Pdgfrb*^flox/flox^ mice on postnatal day 2, and the mice were genotyped at 4–5 weeks old. Mice were fed 60 kcal% HFD (D12492; Research Diet, New Jersey, U.S.A) from 4 weeks old. At 6 weeks old, tamoxifen (Cayman, 2.25 mg per 10 g body weight) was orally administered to *Pdgfrb*^flox/flox^ and Cre^ERTM^/*Pdgfrb*^flox/flox^ mice for 5 consecutive days to produce FL and KO. Mice were euthanized by cervical dislocation at 12 and 20 weeks old after an overnight fast (Figs. 1A and 3A, and 4A). Mice were housed under a 12:12-h light-dark cycle (lights on at 7:00) in a temperature-controlled colony room (20–26 ℃). All animal experiments reported in this study were approved by the Committee of Animal Experiments and Gene Experiments at the University of Toyama (A2017PHA-1, A2018PHA-3, G2013PHA-7, and G2018PHA-2), and were conducted in accordance with ARRIVE guidelines^43^ and our Regulations of Animal Experimentation Handling at University of Toyama.

### Flow cytometry and isolation of liver cells

Male C57BL/6J mice maintained on a regular diet were used as controls for dMASH. The isolation of hepatocytes and hepatic NPCs and methods for a flow cytometric analysis were previously^44,45^. In brief, livers were minced and digested with collagenase in RPMI at 37℃ for 60 min. Samples were passed through a 190-µm nylon mesh, centrifuged at 600 ×g at 4℃ for 8 min, and the supernatants were discarded. Samples were then mixed with 33% Percoll (GE Healthcare), centrifuged at 800 ×g at 4℃ for 30 min, and hepatocytes and NPCs were isolated. The latter cell fraction was hemolyzed with lysing buffer at 4℃ for 15 min, washed twice with FACS buffer consisting of 1% BSA in FACS Sheath Fluid (BD Bioscience, New Jersey, U.S.A), followed by filtration through a 190-µm nylon mesh. Samples were stained with fluorochrome-conjugated antigen-specific antibodies or their isotype control antibodies on ice for 30 min after blocking with the anti-CD16/CD32 antibody on ice for 10 min. After washing once with FACS buffer, cell suspensions were stained with 7AAD just before sorting. The total amount of liver NPCs was analyzed by FACSAria II (BD Biosciences). CD45 + F4/80^hi^CD11b^lo^ cells, CD45^+^F4/80^lo^CD11^hi^ cells, CD45^−^CD31^+^ cells, and CD45^−^Pdgfrβ^+^ cells in liver NPCs were isolated as KCs, infiltrated MFs, ECs, and HSCs, respectively. The actual numbers and percentages of these cells in living cells, excluding cell doublets, were analyzed by FACSDiva 6.1.2 (BD Bioscience) or FCS Express4.0 (De Novo Software, California, U.S.A.). The gating of each cell is shown in Fig. 1B. Antibodies used for flow cytometry are listed in Table 1.

**Table 1:** List of antibodies for flow cytometry.

Antibody	Conjugate	Isotype	Supplier	Identifier (Cat #)	Dilution rate
7AAD			eBioscience	00-6993-50	1:200
CD16/CD32			BD Bioscience	553,141	1:100
CD11b	FITC	Rat IgG2b	BioLegend	101,205	1:100
CD31	PE	Rat IgG2a	Miltenyi Biotec	130-119-653	1:20
CD140b	PE-Vio770	Rat IgG2a	Miltenyi Biotec	130-105-118	1:800
CD45	APC	Rat IgG2a	Tonbo	20-0451-U025	1:100
F4/80	APC/Cy7	Rat IgG2a	BioLegend	123,118	1:100
Isotype control	FITC	Rat IgG2b	BD Bioscience	553,988	1:133.3
Isotype control	PE	Rat IgG2a	Tonbo	50-4321-U100	1:20
Isotype control	PE-Vio770	Rat IgG2a	Miltenyi Biotec	130-102-647	1:800
Isotype control	APC	Rat IgG2a	Tonbo	20-4031-U100	1:100
Isotype control	APC/Cy7	Rat IgG2a	BD Bioscience	400,523	1:100

### scRNA-Seq data analysis using a public human dataset

The expression of PDGF isoforms and related genes in the livers of healthy subjects and cirrhosis patients was analyzed using a publicly available dataset (ArrayExpress database, Accession Number: E-MTAB-10553). To minimize differences in sampling methods and batch effects, we analyzed only data collected by Ramachandran et al. Clinical data on subjects and sampling method details were previously reported^14^, and data preprocessing, including cell annotations, is described in the literature^16^. Seurat v4.4.0 was used for analyses.

### Histological analysis, immunohistochemistry, and evaluation of hepatic tumor sizes

Overnight fasted mice were anesthetized and transcardially perfused with saline. The liver was isolated and divided into two parts; the right lateral and right median lobes, which were stocked for microRNA and gene expression analyses, and the left lateral, left median, and caudate lobes of the liver, which were immersion-fixed in 4% paraformaldehyde overnight and then embedded into paraffin. To analyze the numbers and sizes of liver tumors, the entire left lateral, left median, and caudate lobes of the liver from 20-week-old mice were anchored in a 20% agarose gel, cut into two-mm-thick sections, and embedded into paraffin. Six-micrometer-thick sections were then prepared and used for H&E and Sirius-Red staining^46,47^. H&E sections of the livers of 12-week-old mice were evaluated by NAS^21^ under the guidance of a pathologist. For immunohistochemistry, paraffin-embedded liver sections from 20-week-old FL and KO mice were incubated with rabbit anti-AFP antibody (A0008, DACO-Agilent, California, U.S.A.), rabbit anti-c-Myc (ab32072, Abcam, Cambridge, U.K.), and rabbit anti-MCM7 antibody (11225-1-AP, Proteintech) followed by incubation with an anti-rabbit IgG antibody (Nichirei, Tokyo, Japan), and counterstaining with DAB. Photomicrographs were captured using a BZX800 (Keyence, Osaka, Japan).

### RNA isolation and real-time PCR

Total RNA in the liver, hepatocytes, and each cell fraction obtained by flow cytometry was purified using TRIsure (Nippon Genetics, Tokyo, Japan). Liver samples were collected en bloc unless otherwise noted. For the analysis of non-tumor and tumor liver tissues from 20-week-old mice, samples were taken separately from the right and right median lobes of the liver. For mRNA quantification, cDNA was synthesized using the ReverTra Ace qPCR RT Master Mix with gDNA Remover (Toyobo Co, Osaka, Japan). For miRNA quantification, first strand synthesis was performed using the miRNAssay qPCR RT Master Mix following the manufacturer’s protocol (Toyobo). The reaction mixture included stem-loop RT-primers specific for the target miRNA or U6 small nuclear RNA (snRNA). mRNA expression was analyzed by real-time PCR using SYBR Premix Ex Taq (Takara Bio, Kusatsu, Japan) on a Mx3000/3005P (Agilent, California, U.S.A). miRNA expression was analyzed by real-time PCR using Thunderbird Next SYBR qPCR (Toyobo) for miRNA on a CronoSTAR 96 system (Takara). The sequences of the primers and RT primers used are listed in Table 2. The expression level of the target genes was normalized to *Rn18s* for mRNA^48^ or U6 for miRNA, respectively.

**Table 2:** List of primers used in the present study.

Gene	Forward	Reverse
Rn18s	GTAACCCGTTGAACCCCATT	CCATCCAATCGGTAGTAGCG
aSma	ACCAACTGGGACGACATGGAA	TGTCAGCAGTGTCGGATGCTC
Afp	TGGTTACACGAGGAAAGCCC	AATGTCGGCCATTCCCTCAC
Cd9	TGCTGGGATTGTTCTTCGGG	GCTTTGAGTGTTTCCCGCTG
Cdkn1a	GTGGCCTTGTCGCTGTCTT	GCGCTTGGAGTGATAGAAATCTG
Col1a1	GCTCCTCTTAGGGGCCACT	CCACGTCTCACCATTGGGG
Emr1	CTTTGGCTATGGGCTTCCAGTC	GCAAGGAGGACAGAGTTTATCGTG
Gpr55	AGTCCATATCCCCACCTTCC	AGGGAGAGCACCAGCAGTAA
Hgf	CTTTGGCTATGGGCTTCCAGTC	GACCAGGAACAATGACACCA
Il1b	TCCAGGATGAGGACATGAGCAC	GAACGTCACACACCAGCAGGTTA
Mcm7	GAGACCTACCAGCCAATCCA	CCCACAGGAACTTGGTCACT
Myc	TGACCTAACTCGAGGAGGAGCTGGAATC	AAGTTTGAGGCAGTTAAAATTATGGCTGAAGC
Pdgfa	GACGGTCATTTACGAGATAC	TCTTCCTGACATACTCCACT
Pdgfb	CCCACAGTGGCTTTTCATTT	GTGAACGTAGGGGAAGTGGA
Pdgfc	AGGTTGTCTCCTGGTCAAGC	CCTGCGTTTCCTCTACACAC
Pdgfd	CAGTCTTCTTCCCACGATGC	ATGTCCAGGCTCAAACTTCA
Pdgfra	TGGCATGATGGTCGATTCTA	CGCTGAGGTGGTAGAAGGAG
Pdgfrb	AGGACAACCGTACCTTGGGTGACT	CAGTTCTGACACGTACCGGGTCTC
Pten	ATCACCTGGATTACAGACCCGT	CCACAAACTGAGGATTGCAAGTT
Tgfb	GTGTGGAGCAACATGTGGAACTCTA	TTGGTTCAGCCACTGCCGTA
Tnfa	AAGCCTGTAGCCCACGTCGTA	GGCACCACTAGTTGGTTGTCTTTG
Timp1	CATCACGGGCCGCCTA	AAGCTGCAGGCACTGATGTG
snRNA-U6 -RT	AACGCTTCACGAATTTGCGT	
nRNA-U6	CTCGCTTCGGCAGCACA	AACGCTTCACGAATTTGCGT
miR-1291-RT	GTTGGCTCTGGTGCAGGGTCCGAGGTATTCGCACCAGAGCCAACAACTGC	
miR-1291	GTTTTTGGATGGCTCTTACTGAAGA	GTGCAGGGTCCGAGGT

### Serum biological analysis

Random-fed blood glucose levels were measured once a week using a free-style blood glucose sensor (Abbot Japan, Tokyo, Japan). Serum AST and ALT levels were analyzed with the Transaminase CII test kit (FUJIFILM Wako Co., Osaka, Japan).

### Profiling of miRNA expression

Total RNA was extracted from the livers of 20-week-old FL and KO using miRNeasy Mini kits (Qiagen, Valencia, CA) according to the manufacturer’s instructions. cDNA, generated with a miScript II RT Kit (Qiagen), was used as a template for the miRNA expression analysis (*n* = 5/group) using the GeneChip miRNA 4.0 array (Thermo Fisher Scientific, Tokyo, Japan). Data were analyzed using Transcriptome Analysis Console software 4.0 (Thermo Fisher Scientific) and GeneSpring Ingenuity Pathways Analysis software (Qiagen, Tokyo, Japan), with an adjusted cut-off P-value of 0.05 and a fold-change threshold of 1.2.

### Statistical analysis

Data are expressed as means ± standard error of the mean (SEM). Statistical analyses were performed using the Student’s *t*-test between two groups or a one-way ANOVA with Tukey’s test for multiple comparisons by GraphPad Prism 9 software (GraphPad Software Inc., USA). *p < 0.05 and **p < 0.01 indicated significant differences.

## Electronic supplementary material

Below is the link to the electronic supplementary material.

Supplementary Material 1

## Data Availability

The datasets generated and/or analyzed in this study will be available on reasonable request to the corresponding author.
