# Supplementary material for: Deletion of platelet-derived growth factor receptor β suppresses tumorigenesis in metabolic dysfunction-associated steatohepatitis (MASH) mice with diabetes
Source: Sci Rep. 2024 Oct 11;14:23829. doi: 10.1038/s41598-024-75713-6 (PMC11470010; doi:10.1038/s41598-024-75713-6)
Supplement: Supplementary file 1 — Supplementary Material 1 [file 41598_2024_75713_MOESM1_ESM.pdf]

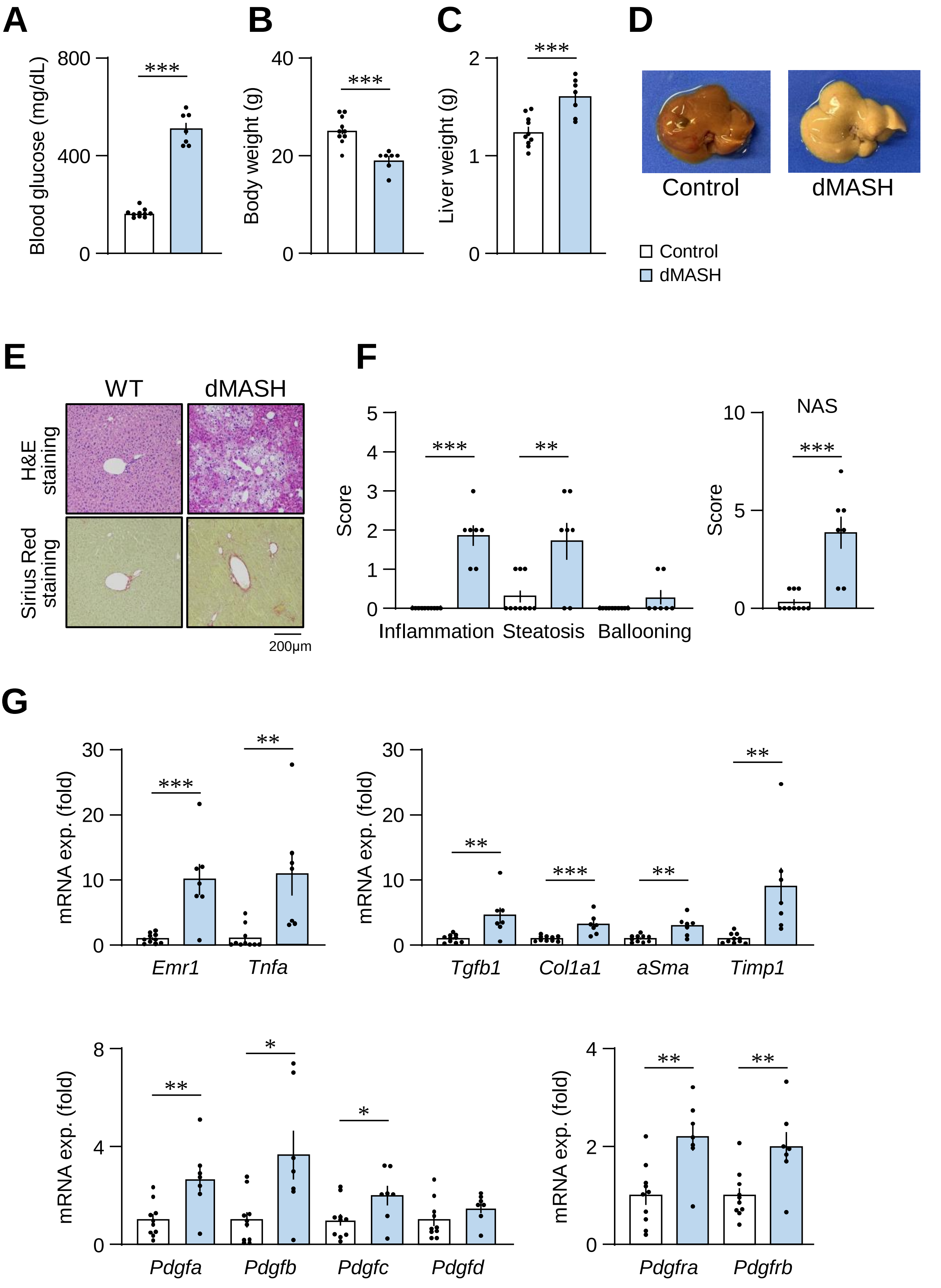

Supplementary Fig. S1. Phenotypes of dMASH in C57BL/6J at 12 weeks old (related to Fig. 1).
(A-C) Blood glucose levels and body and liver weights in control and dMASH. (D) Macroscopic appearance of the whole liver. (E) Representative photomicrograph of liver sections in H&E and Sirius-Red staining. Scale bar = 200 μm. (F) Histological evaluation by NAS. (G) mRNA levels of genes related to chronic inflammation, fibrosis, and the *Pdgf* family. Data are presented as the mean ± SEM (Control, n=10; dMASH, n=7). *p <0.05, **p <0.01, and ***p <0.001.

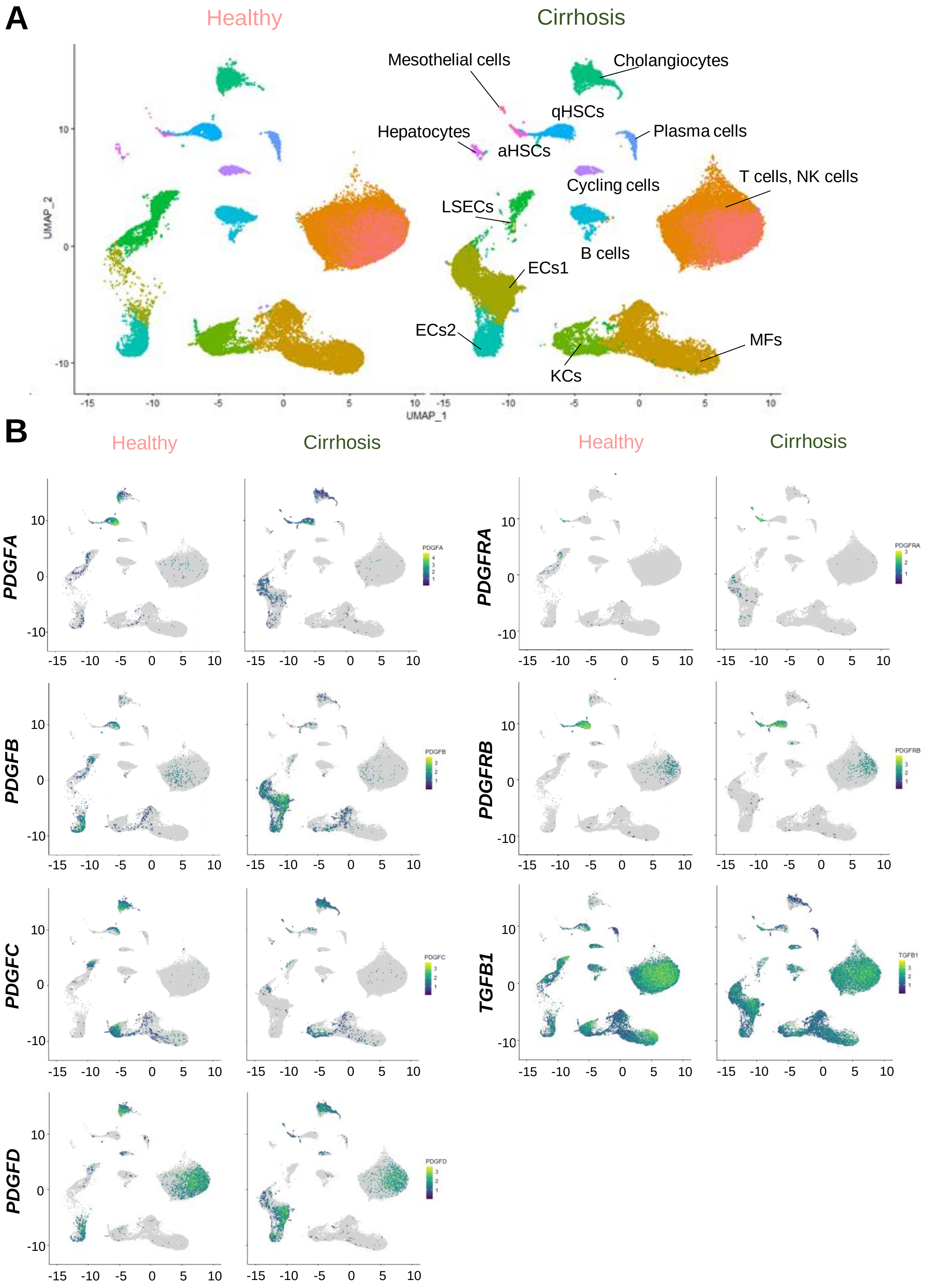

Supplementary Fig. S2. Expression profiles of the PDGF family in healthy subjects and cirrhosis patients by feature plots (related to Fig. 2).

The expression profiles of *PDGF*-related genes by cell type were re-analyzed using publicly available scRNA-Seq data. (A) UMAP projection of all liver cells split by healthy subjects and cirrhosis patients. (B) Cell type-specific expression profiles displayed by feature plots. Results are displayed using violin plots in Fig. 2.

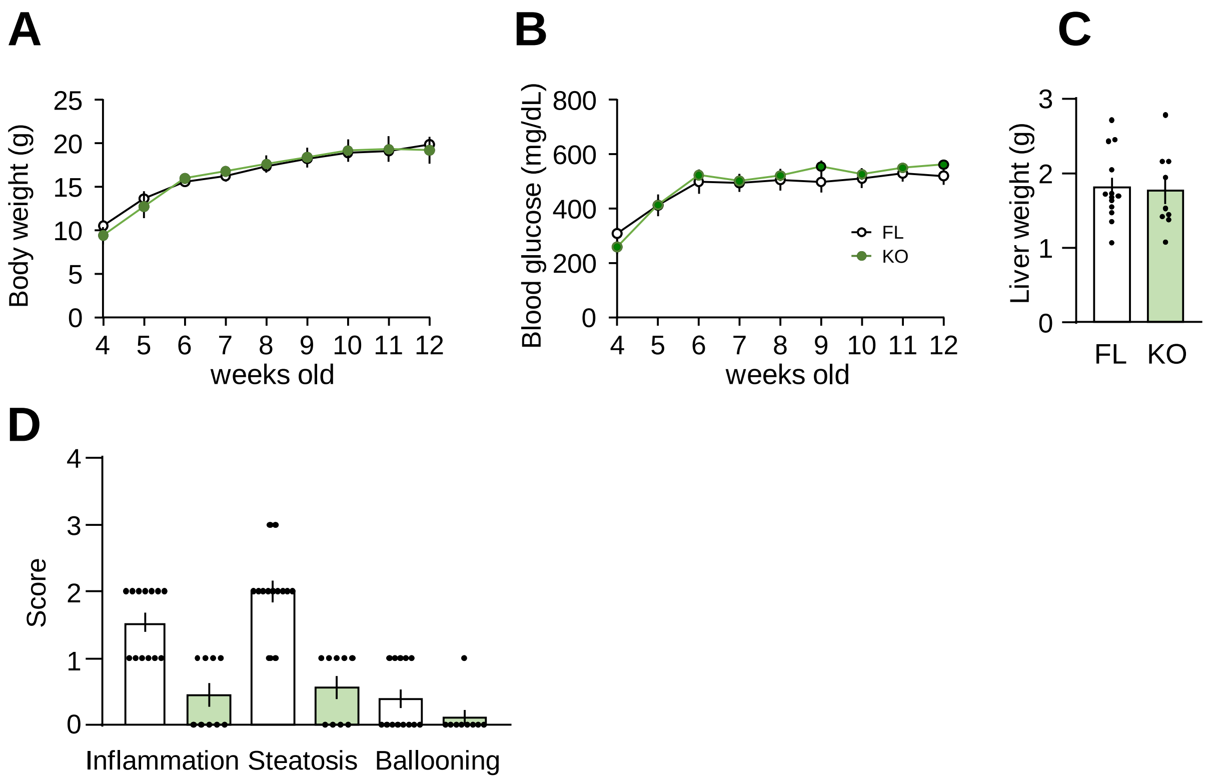

Supplementary Fig. S3. Basic characteristics of dMASH in FL and KO dissected at 12 weeks old (related to Fig. 3).

(A, B) Changes in body weights and casual blood glucose levels in FL and KO dMASH. (C) Liver weights at dissection. (D) Histological evaluation of inflammation, steatosis, and ballooning for NAS. The total NAS score is shown in Fig. 3B. Data are presented as the mean ± SEM (FL, n=13; KO, n=9).

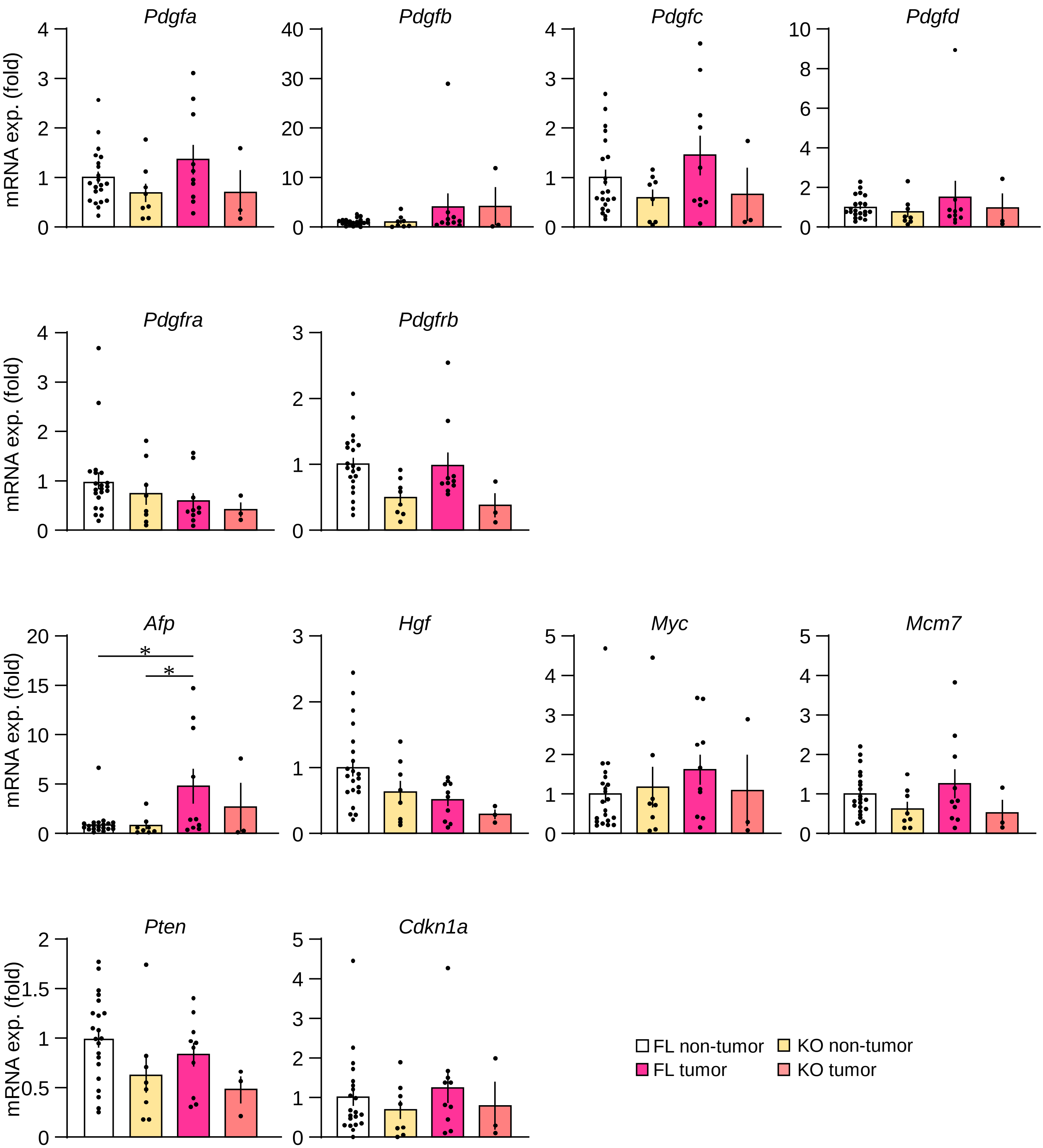

Supplementary Fig. S4. Impact of the deletion of PDGFRβ on mRNA expression in non-tumor and tumor lesions in dMASH livers at 20 weeks old (related to Fig. 4).

The mRNA levels of *Pdgf*- and tumorigenesis-related genes in non-tumor and tumor lesions in FL and KO livers (non-tumor FL, n=21; non-tumor KO, n=8; tumor FL, n=10, tumor KO, n=3). Data are presented as the mean ± SEM. *$p<0.05$ by a one-way ANOVA with Tukey's test.

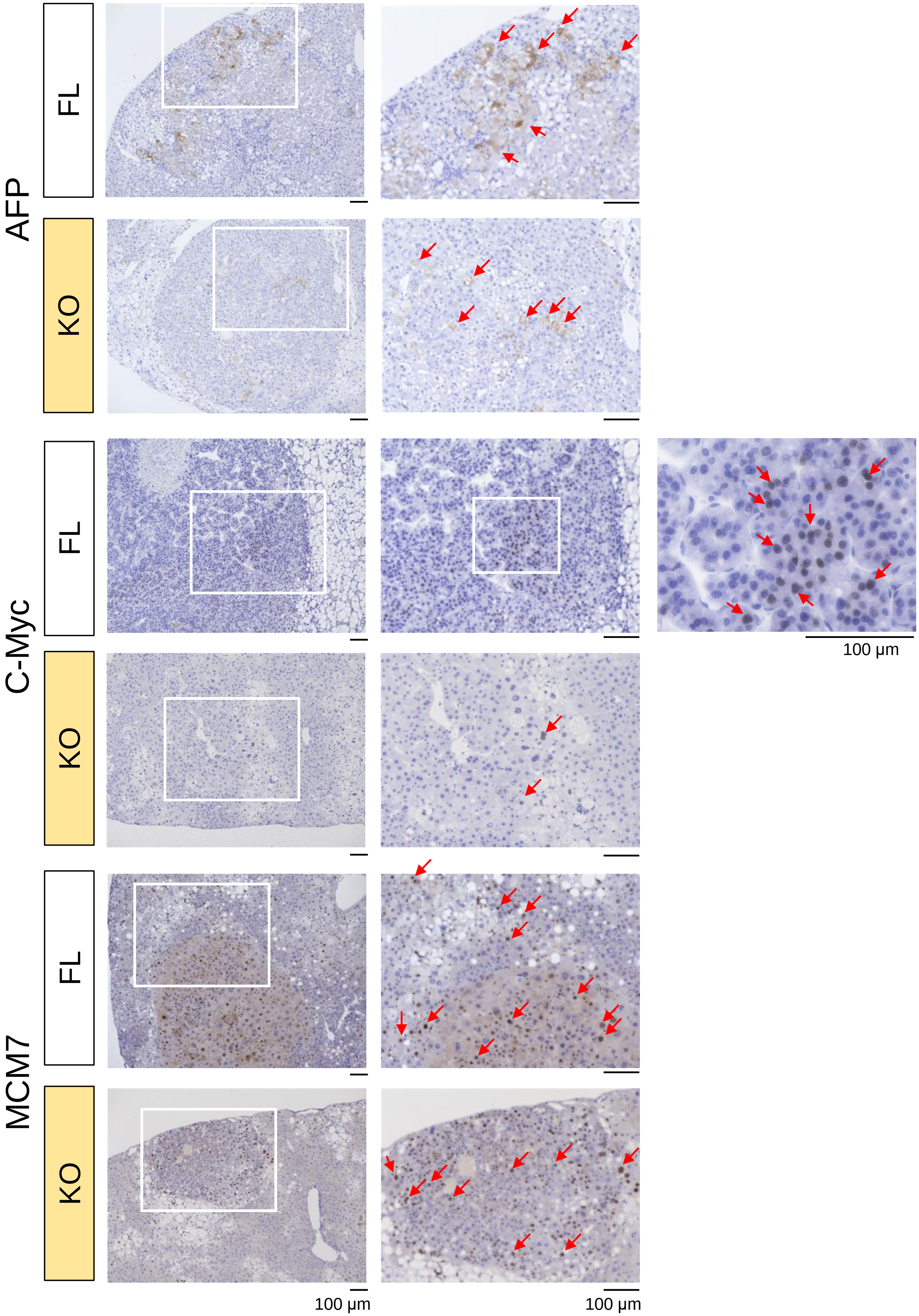

Supplementary Fig. S5. Immunohistochemical analysis of FL and KO liver (related to Fig. 4).
Representative photomicrograph of well-stained liver sections immunostained with anti-AFP, anti-c-Myc, or anti-MCM7 antibodies. A close-up of the area within the white box is displayed on the right. Arrows indicate well-stained cells. Scale bar = 100 µm.

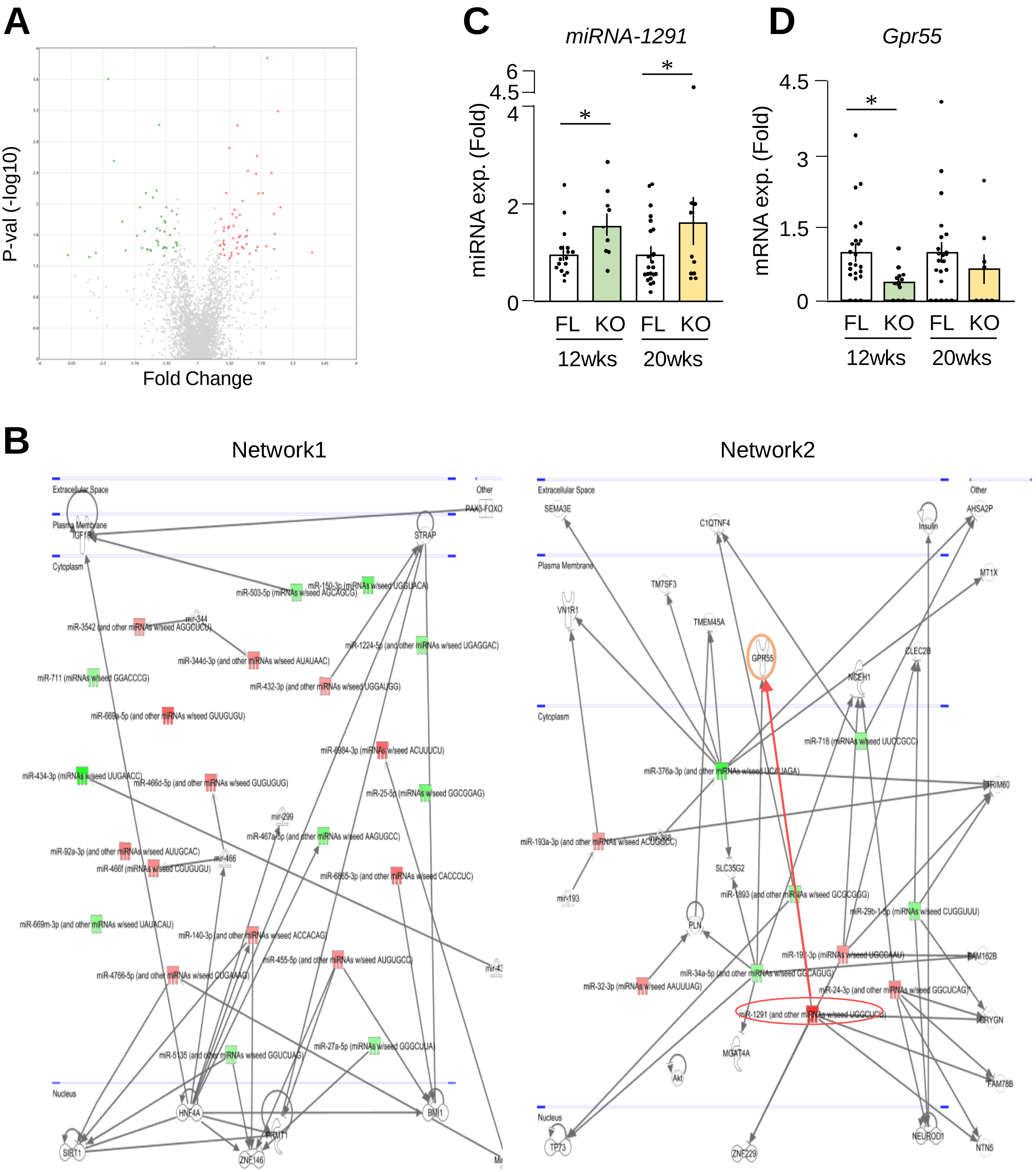

Supplementary Fig. S6. Differential miRNA expression and a functional network pathway analysis between 20-week-old FL and KO dMASH (related to Fig. 4).

Total RNA extracted from non-tumor regions of the livers of 20-week-old FL and KO was analyzed using the GeneChip miRNA 4.0 array. (A) Differential miRNA expression between FL and KO is shown in volcano plot. miRNAs with significantly higher or lower expression (p<0.05) in KO than in FL are indicated by a red or moss-blue dot, respectively. (B) Networks 1 and 2 in the Ingenuity Pathway Analysis (IPA) of miRNAs that differed between KO and FL. Predicted potential interactions of miRNAs and downstream target molecules in each network are shown. (C) The expression of miRNA-1291 in 12 and 20-week old FL and KO livers (12w: FL, n=13, KO, n=9; 20w: FL, n=19, KO, n=8). (D) The expression of *Gpr55*, a target of miRNA-1291 identified by the IPA network analysis, in 12- and 20-week-old FL and KO livers (12w: FL, n=21, KO, n=8; 20w: FL, n=21, KO, n=8). Data are presented as the mean ± SEM. *p<0.05.
